# Pulcherriminic acid relay; a *Bacilli* route to attack pathogens

**DOI:** 10.3389/fcimb.2025.1740921

**Published:** 2026-01-20

**Authors:** Ramya Srinivasan, Chaitany Jayprakash Raorane, Tamil Selvam Saravanan, Romain Briandet, Satish Kumar Rajasekharan

**Affiliations:** 1 Department of Biotechnology, School of Bioengineering, SRM Institute of Science and Technology, Kattankulathur, Tamil Nadu, India; 2 School of Chemical Engineering, Yeungnam University, Gyeongsan, Republic of Korea; 3 Université Paris-Saclay, INRAE, AgroParisTech, Micalis Institute, Jouy-en-Josas, France

**Keywords:** antibiofilm, *B. subtilis*, biofilm, fur system, pulcherrimin, *S. aureus*

## Abstract

Pulcherriminic acid (PA) relay is a recently discovered phenomenon in which the *Bacillus subtilis* employs branching biofilms to relay the antimicrobial pigment, pulcherriminic acid towards the pathogen. PA interacts with the free iron in the environment to form the reddish-pink pigment, pulcherimin, which subsequently accumulates on the pathogen depriving them of the essential iron. In *Staphylococcus aureus*, the ferric uptake regulator (Fur) system plays a vital role in maintaining iron homeostasis, virulence, and biofilm formation. The perspective article discusses the plausible mechanistic insights on the impact of PA relay in hampering the Fur system. Taken together, these findings highlight PA and PA-producing *Bacillus* species as a promising alternative for mitigating drug resistant *S. aureus* infections.

## Role of iron in microbial pathogenesis

Iron is a crucial micronutrient essential for all microorganisms, as it facilitates their metabolic processes, enzymatic reactions, respiration, and DNA synthesis, and also serves as a cofactor in enzymes that mediate redox reactions (Hammer and Skaar, 2011; Li et al., 2021; Sheldon et al., 2016). In environments where iron is scarce, such as within human hosts or in processed food, bacteria employ sophisticated siderophore–mediated uptake systems and heme acquisition pathways to survive and establish biofilms (Saha et al., 2025; Sheldon et al., 2016; Weinberg, 2004). Robust Biofilm formation represents a major virulence strategy, enabling pathogens to persist in hostile environments and tolerate antimicrobial interventions (Charron et al., 2025), is tightly linked to iron levels, where excess iron promotes aggregation, while scarcity results in motility and reduced adherence (Conroy et al., 2019; Sheldon et al., 2016). The competition for iron between the host and pathogen has significantly influenced the evolution of both groups in this complex relationship. Hosts have developed nutritional immunity mechanisms to sequester iron and limit its availability to invading pathogens, and bacteria have countered with increasingly sophisticated iron acquisition systems (Hood and Skaar, 2012). Even small shifts in iron availability within food environments or on processing surfaces can significantly alter microbial dominance, influencing both product safety and shelf–life (Carrascosa et al., 2021). Consequently, limiting iron availability can effectively impair bacterial growth and virulence (Sheldon et al., 2016), making iron metabolism a valuable target for novel antibiofilm strategies.

## *Staphylococcus aureus* and iron regulation

*Staphylococcus aureus* is a notorious food-borne pathogen whose virulence is intricately fastened to iron acquisition and biofilm formation (Lin et al., 2012, Lin et al., 2012; Paiva et al., 2021; Van Dijk et al., 2022). This bacterium is a major concern in food safety due to its capacity to spoil food products and cause food poisoning. Its virulence is driven by multiple factors, including enterotoxin production and strong biofilm formation, which enhance its tolerance to antimicrobials (Fetsch and Johler, 2018; Paiva et al., 2021; Phan et al., 2025; Sergelidis and Angelidis, 2017). *S. aureus* relies on multiple iron-acquisition strategies: the ferric-uptake regulator (Fur) controls high-affinity siderophores (staphyloferrin A/B), heme uptake via the *Isd* pathway, and the *Cnt* system for nickel/cobalt. These pathways are up-regulated during iron limitation (Batko et al., 2021; Ghssein and Ezzeddine, 2022). Iron availability significantly impacts *S. aureus* biofilm formation, toxin production, and overall virulence, highlighting the ferric uptake regulator (Fur) system as an attractive target for antimicrobial intervention (Lin et al., 2012; Van Dijk et al., 2022).

## The Fur system

The siderophores staphyloferrin A and B are synthesized via nonribosomal peptide synthetase-independent pathways. The efficient scavenge extracellular ferric iron, which is subsequently imported through ATP-binding cassette transporters, specifically *htsABC* for staphyloferrin A, as well as *sirABC* for staphyloferrin B, frequently with the assistance of the ATPase FhuC. Regulation of these genes is mediated by the ferric uptake regulator protein (*Fur*), which regulates siderophore biosynthesis as well as transport to occur mostly under conditions of iron deprivation. Current studies suggest that reductases, including *IruO* as well as *NtrA*, participate in reducing as well as releasing iron in its soluble form from siderophore-iron complexes intracellularly. Furthermore, *SbnI*, a heme-binding regulatory protein, links siderophore biosynthesis with intracellular heme sensing, revealing a complex interplay between heme- and siderophore-mediated iron metabolism (Beasley and Heinrichs, 2010; Conroy et al., 2019; Hammer and Skaar, 2011). The concept of iron deprivation as an antimicrobial tactic has gained attention due to the integral role of iron in biofilm development and virulence expression (Sheldon et al., 2016). Iron restriction strategies aim to destabilize bacterial growth through “nutritional immunity” or exogenous chelation. Host systems naturally deploy proteins, such as transferrin and lactoferrin to deprive pathogens of this essential nutrient (Hammer and Skaar, 2011). Artificial chelating agents, including EDTA, β-thujaplicin, or deferoxamine, mimic this mechanism and effectively inhibit biofilm development by destabilizing iron homeostasis (Bereswill et al., 2022; Saha et al., 2025; Soldano et al., 2020). However, synthetic chelators often pose biocompatibility and safety concerns in food applications. This has shifted scientific interest toward microbial molecules with self-regulated eco-friendly, and food-grade iron chelating properties.

## Pulcherriminic acid relay; a *Bacilli* route to attack pathogens

**Figure 1:**
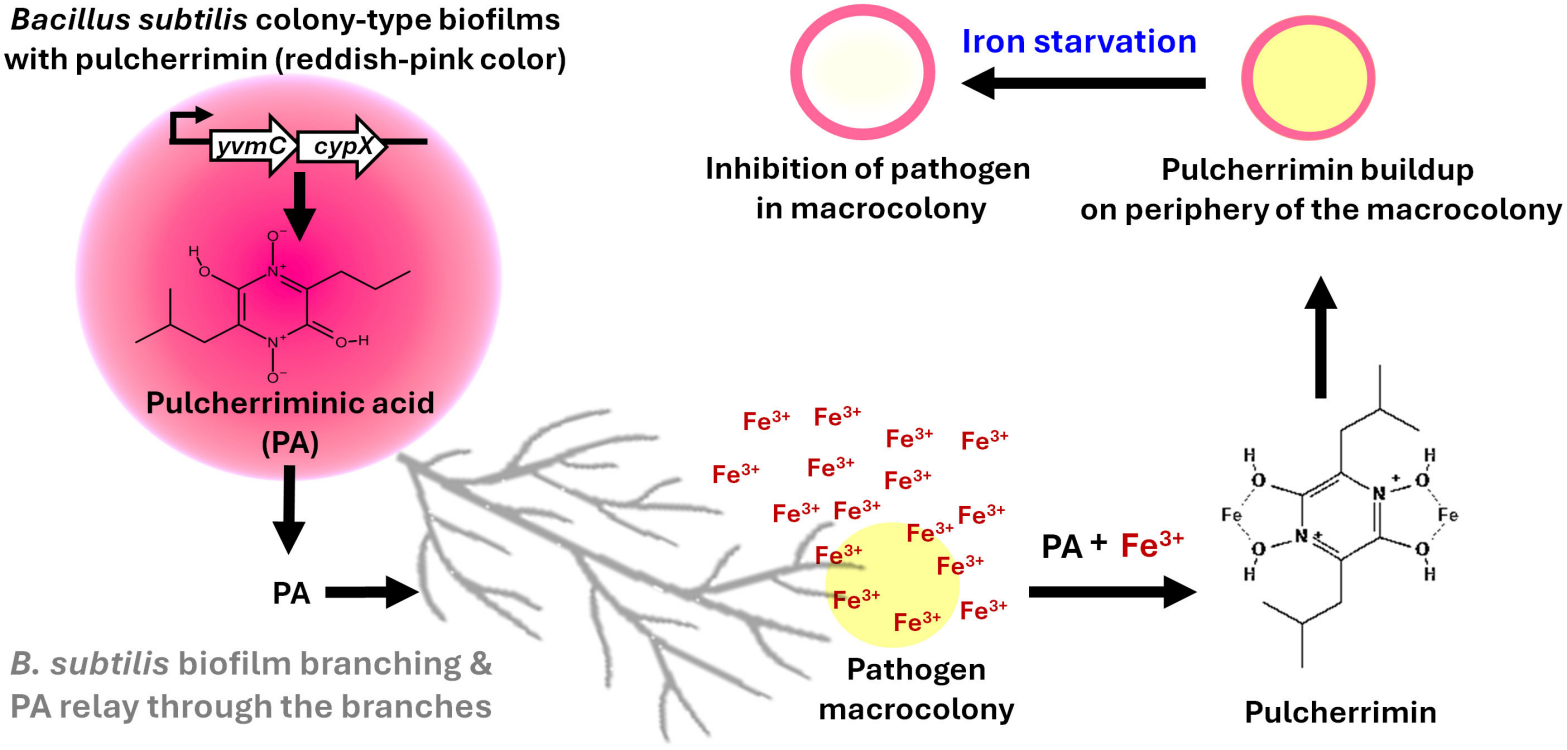
The established concept of pulcherriminic acid relay by Bacillus subtilis for control of pathogens. *B. subtilis* colony-type biofilm produces pulcherriminic acid (PA) which is relayed through branching structures toward the neighbouring pathogen. PA chelates environmental ferric iron (Fe³^+^) in the vicinity of the pathogen, forming the insoluble reddish-pink pigment pulcherrimin at the periphery of the pathogen macrocolony. This sequestration of Fe³^+^ causes iron starvation within the pathogen, leading to growth inhibition.

**Figure 2:**
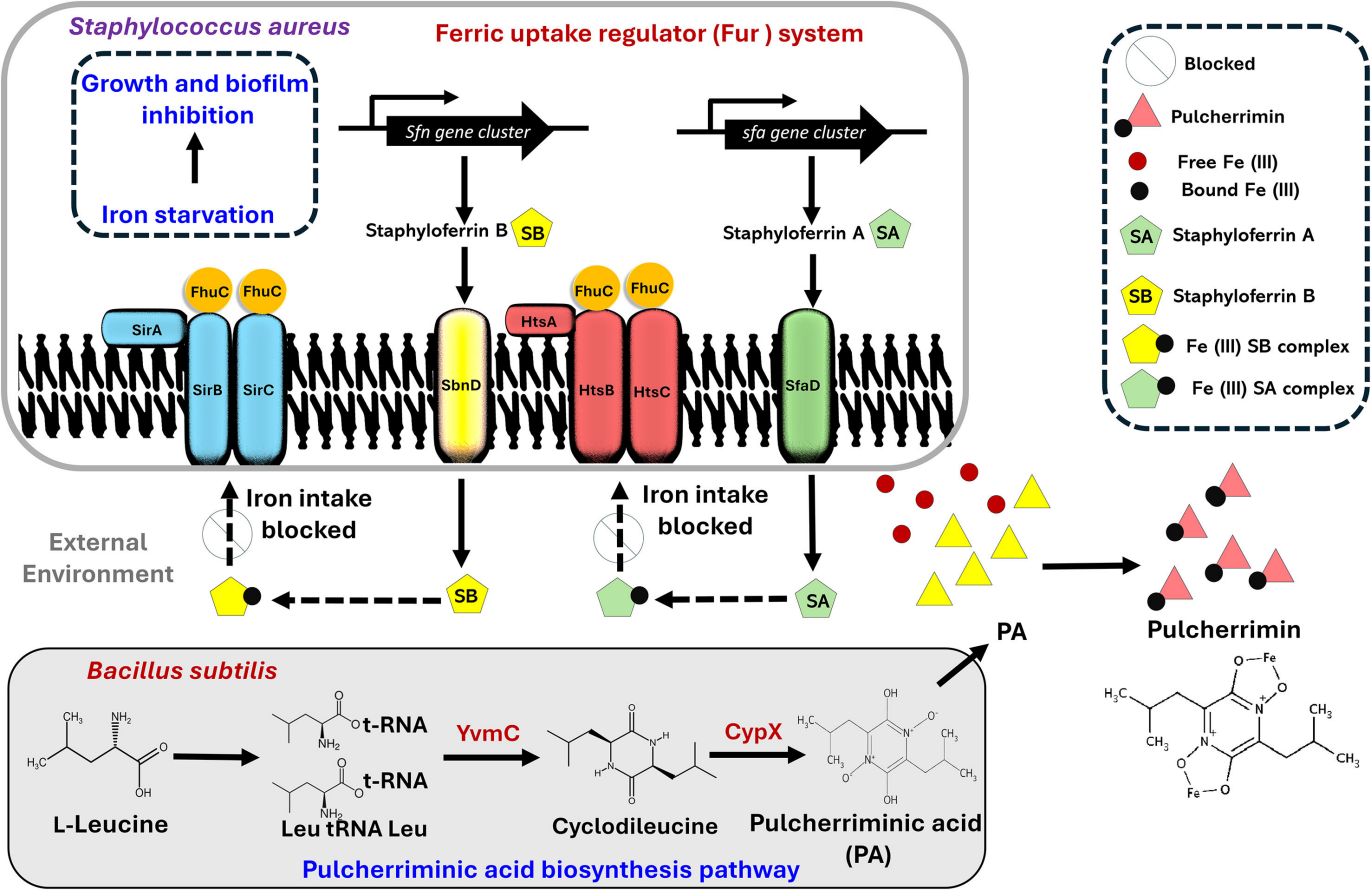
Proposed model depicting the interaction between *Staphylococcus aureus* and *Bacillus subtilis*. *S. aureus* uses the Fur system to acquire Fe (III) from the external environment via the formation of siderophore-iron (staphyloferrin A-Fe (III) and staphyloferrin B-Fe (III)) complexes. During competition with *B subtilis* (a strain known to secrete pulcherriminic acid (PA)), PA diffuses to the external environment, hijacks the free iron and precipitates as pulcherrimin, thus limiting the availability of iron to form the siderophore-Fe (III) complexes, thereby leading to iron starvation and inhibition of *S. aureus*.

Pulcherriminic acid (PA) has emerged as a promising cyclic dipeptide with potent activity against several pathogens. The pigment is synthesized intracellularly as PA by the action of two key enzymes, *YvmC* and *CypX*, in *B. subtilis* cells, and PA diffuses out of the cell and complexes with free iron to form pulcherrimin (Angelini et al., 2023; Arnaouteli et al., 2019) (**Figures 1**, **2**). Recent studies have shed light on its mechanism of action and relay, underscoring its therapeutic potential (Angelini et al., 2023; Arnaouteli et al., 2019; Charron-Lamoureux et al., 2023). Notably, *B. subtilis* strategically relays the pulcherrimin precursor, pulcherriminic acid, within developing antagonistic biofilms to counteract pathogens (**Figure 1**). The mechanism of PA relay was first shown by our group in *C. albicans* (Rajasekharan et al., 2025). This targeted delivery system ensures localized iron chelation precisely at the sites where the pathogen attempts to establish itself, effectively halting its growth and suppressing morphological switching (Rajasekharan et al., 2025). The core mechanism behind this involves the disruption of *C. albicans* iron uptake systems. By strongly binding Fe³^+^ ions, pulcherrimin induces a state of iron starvation. The “targeted relay” delivery mechanism offers a novel framework for optimizing pulcherrimin or precursor-based formulations in future therapeutic strategies. This finding paves the way for exploiting iron acquisition mechanisms as a therapeutic agent against other pathogens, notably *S. aureus*, which also reveals a similar phenomenon (data not shown). We believe that this new interaction and pigment relay is a competitive ecological strategy by *B. subtilis* to suppress competitors within mixed microbial communities.

## Proposed model to block the Fur system

*S. aureus* uses the Fur system to acquire iron from the external environment via the formation of siderophore-iron complexes. Fur systems rely on high-affinity chelators staphyloferrin A and B, which are also synthesized by non-ribosomal peptide synthetase-independent routes. The siderophores scavenge extracellular ferric iron and transport via ATP-binding cassette transporters, specifically HtsABC for staphyloferrin A, as well as SirABC for staphyloferrin B, with the help of the ATPase FhuC. As illustrated in **Figure 2**, we suggest that the pulcherriminic acid secreted by *B. subtilis* is released into the external environment, where it binds the free iron to form the reddish pink pulcherrimin, thus depriving *S. aureus* of essential iron required for the formation of siderophore-iron complexes. Overall, the inability to form iron-siderophore complexes prevents any iron intake into the *S. aureus* cell, thus compromising the overall mechanistic framework, ultimately compromising cellular metabolism and viability.

PA represents the primary bioactive molecule responsible for antimicrobial activity in *Bacillus* sp*ecies*, functioning through iron chelation and subsequent nutrient deprivation. The antimicrobial efficacy of this system is therefore highly context-dependent and shaped by competitive iron-acquisition strategies within microbial communities. For instance, *B. subtilis* can partially overcome PA-mediated iron sequestration through the production of the high-affinity siderophore bacillibactin, enabling iron recovery from pulcherrimin complexes, whereas competing pathogens lacking comparable metallophore systems are more susceptible to iron starvation (Charron-Lamoureux et al., 2023). In addition, PA exhibits pronounced photosensitivity and undergoes light-induced degradation, a process shown to dynamically regulate iron availability and biofilm development in *B. subtilis* (Kobayashi et al., 2025). This property has significant implications for applied use, as light exposure during food processing, storage, or transport may reduce PA stability and limit the persistence of its antimicrobial effect. Consequently, effective deployment of PA-based biocontrol strategies must account for both microbial competition for iron and environmentally driven modulation of PA activity.

## Conclusion

In conclusion, the use of *Bacillus-*derived pulcherriminic acid in food safety shows strong potential. As a naturally occurring, broad-spectrum antimicrobial, PA effectively targets iron-dependent pathogens and can complement conventional preservatives and antibiotics. By limiting iron availability to competing microorganisms, PA functions as a promising biocontrol agent. Its natural origin, stability, and compatibility with food systems make it an appealing candidate for sustainable and safe food protection strategies.
